# Acute kidney injury; the focus of world kidney day in 2013

**Published:** 2013-09-01

**Authors:** Mohammad-Reza Ardalan, Hamid Nasri

**Affiliations:** ^1^Chronic Kidney Disease Research Center, Tabriz University of Medical Sciences, Tabriz, Iran; ^2^Department of Nephrology, Division of Nephropathology, Isfahan University of Medical Sciences, Isfahan, Iran

**Keywords:** World kidney day, Diabetic nephropathy, Renal failure

**Implication for health policy/practice/research/medical education:**

In 2013 World Kidney Day (WKD) will focus on acute kidney injury (AKI) as a global health alert. This day is a yearly event prearranged both by the International Society of Nephrology (ISN) and the International Federation of Kidney Foundations (IFKF). In this year, the WKD committee aimed to alert the worldwide increase in AKI. AKI is defined by sudden decrease in kidney function by decrease in glomerular filtration rate, followed by accumulation of nitrogenous waste products and the incapability to maintain fluid and electrolyte homeostasis, which usually consistent by decrease in urine output and various clinical presentations.

Again, the World Kidney Day in March 14 reached. In this year World Kidney Day (WKD) will focus on acute kidney injury (AKI) as a global health alert. Indeed this is the 8th WKD, will be celebrated (1). This day is a yearly event prearranged both by the International Society of Nephrology (ISN) and the International Federation of Kidney Foundations (IFKF) (1-3). In this year, the WKD committee aimed to alert the worldwide increase in AKI (4). AKI is defined by sudden decrease in kidney function by decrease in glomerular filtration rate (GFR), followed by accumulation of nitrogenous waste products and the incapability to maintain fluid and electrolyte homeostasis (5-7), which usually consistent by decrease in urine output and various clinical presentations. This condition is highly associated with increased early and long term mortality and morbidity of these patients. Additionally also there is also a risk of the development of chronic kidney failure subsequently (5-7). In spite of progress in the understanding of pathogenesis of acute kidney dysfunction, we only have an imprecise opinion as to why kidney function deteriorates so dramatically in many patients with acute illness or injury, or why, despite renal replacement therapy, mortality is so high (8,9). While the incidence of AKI has been increasing over time, alongside, the prevalence of chronic kidney failure has also been increasing. Since AKI has long been considered of as a completely reversible disease, nevertheless, over the past several years, a mass of data from experimental animals and humans have been published and pointed out that, AKI more than likely leads to permanent kidney injury as chronic kidney failure (10,11). On the other hand, the percentage of patients existing after AKI has also been increasing over time (4,10,11). Thus, if AKI really increase the risk for chronic kidney failure, then it could imply significant public health concerns with regard to the quantity of persons developing incident chronic kidney failure, progressive chronic kidney failure, end-stage kidney failure (4,10,11). The details why AKI would increase the risk of chronic kidney failure, end-stage kidney failure, and other adverse outcomes not yet fully recognized. Some animal researches imply that AKI can stimulate glomerular and interstitial fibrosis (7-12). Thus, despite the point that AKI is typically reversible in nature, nonetheless, there may be subclinical renal injury that persists and mediates this outcome (7-12). Therefore, an international health strategy is indispensable to diminish the huge growing load of AKI and its complications. In facts, efforts should focused on preventing AKI accompanied by early recognition and treatment, and sufficient follow up to decrease the mortality and the long term incidence of post–AKI chronic kidney failure (4-12). AKI is defined by one of the followings: rise in serum creatinine to ≥1.5 times baseline or rise in serum creatinine by ≥0.3 mg/dl during 48 hours; or urine volume <0.5 ml/kg/h for 6 hours (4-6). Early examination should consisted differentiating, pre-renal and post-renal components from intrinsic renal disease. Biological markers may give early detection of AKI and can help out the differential diagnosis and consideration of prognosis (4-10). Inadequacies in managing have been discovered as contributing factors in the death of many patients with AKI (5-12). In spite of developments in the understanding of the pathogenesis of human AKI, our ability to assess renal function is limited and functional impairment poorly correlates with structural damage to the kidneys (6-11).

Results from a number of investigations have shown that AKI is common, increasing in incidence, and is associated with considerable morbidity and mortality. In the recent study conducted by Aitken et al. on the demographic data of 1577 patients admitted to a teaching medical center during a one month period in UK, found the incidence of AKI at the time of admission was 4.6%. An additional 10.3% developed AKI during the hospital admission. All cause mortality was 4-fold higher among patients with AKI compared with those without. Mortality was meaningfully higher in those patients who developed AKI while an in-patient compared with those with AKI on admission. AKI was unrecognized in 23.5% of patients, two-thirds of whom were discharged without resolving of renal function. They concluded that AKI is common in hospitalized patients and is related to a significant increase in hospital admission and morbidity and mortality (10).

Many common causes of AKI in critically ill patients exist (2-9). Investigations showed that sepsis remains the leading cause of AKI among the critically ill patients accounting for nearly 50% of cases (1-6). Many studies have reported that sepsis-induced AKI is associated with short and long-term risk of death (1-9). In fact recent findings into the pathogenesis of AKI in sepsis are beginning to shift attention from kidney blood flow to inflammation-mediated organ damage (4-12). A diagnostic evaluation can be used to classify acute renal damage as pre-renal, intrinsic renal, or post-renal (5-10). The preliminary workup contains the patient history to find the use of nephrotoxic medications or systemic disease that might cause poor renal perfusion or directly impair renal function (5-10). Protective substances such as N-acetyl-L-cysteine, prostaglandins, allopurinol and various antioxidants can be used. Treatments entails the elimination of post-renal and pre-renal causes of AKI, adjustment of dosages of drugs according to renal status, avoidance of both low arterial pressure and over-hydration, keeping of electrolytic balance, avoiding hyperkalemia and correcting of hyperglycemia and nutritional support (5-10).

## Authors’ contributions

HN and MRA wrote the paper equally.

## Conflict of interests

The authors declared no competing interests.

## Ethical considerations

Ethical issues (including plagiarism, data fabrication, double publication) have been completely observed by the authors.

## Funding/Support

None.
